# Profiling Pre-MicroRNA and Mature MicroRNA Expressions Using a Single Microarray and Avoiding Separate Sample Preparation

**DOI:** 10.3390/microarrays2010024

**Published:** 2013-03-14

**Authors:** Lin Gan, Bernd Denecke

**Affiliations:** Interdisciplinary Centre for Clinical Research Aachen, RWTH Aachen University, Pauwelstr. 30, 52074 Aachen, Germany; E-Mail: lgan@ukaachen.de

**Keywords:** mature microRNA, pre-microRNA, profiling, microarray

## Abstract

Mature microRNA is a crucial component in the gene expression regulation network. At the same time, microRNA gene expression and procession is regulated in a precise and collaborated way. Pre-microRNAs mediate products during the microRNA transcription process, they can provide hints of microRNA gene expression regulation or can serve as alternative biomarkers. To date, little effort has been devoted to pre-microRNA expression profiling. In this study, three human and three mouse microRNA profile data sets, based on the Affymetrix miRNA 2.0 array, have been re-analyzed for both mature and pre-microRNA signals as a primary test of parallel mature/pre-microRNA expression profiling on a single platform. The results not only demonstrated a glimpse of pre-microRNA expression in human and mouse, but also the relationship of microRNA expressions between pre- and mature forms. The study also showed a possible application of currently available microRNA microarrays in profiling pre-microRNA expression in a time and cost effective manner.

## 1. Introduction

MicroRNAs (miRNA) are short conservative endogenous non-coding RNAs with a length of around 22 nucleotides, which have diverse functions [1]. Since its discovery in 1993 [2], miRNA has been recognized as one of the key players in the transcript regulatory network of eukaryotes [3]. Despite intensive scientific research, many miRNAs still need to be explored or validated in different biological or pathological contexts. 

### 1.1. Pre-miRNA, Mature miRNA and miRNA Biogenesis

miRNA genes are transcribed by RNA polymerase II (Pol II) [4] in the nucleus. This process yields long primary transcripts of miRNA (pri-miRNAs). Nuclear RNase III Drosha cleaves pri-miRNA into a precursor of miRNA (pre-miRNA) [5]. Pre-miRNAs are then exported from the nucleus into the cytoplasm by exportin-5 (Exp5) [6]. After further processing by cytoplasmic RNase III Dicer [7,8], mature miRNA is integrated into the miRNA-containing RNA-induced silencing complex (miRISC) [9,10]. miRNA transcription and maturation is a precise controlled and collaborated process [11]. 

### 1.2. Microarray Application in miRNA Expression Profiling

Diverse techniques are available for the purpose of miRNA expression profiling, e.g., Northern blotting [12], dot blotting [13], primer extension analysis [14], RT-qPCR [15] and next generation sequencing (NGS) [16]. RT-qPCR, microarray and NGS are the three most state-of-the-art methods applied in miRNA profiling [17]. RT-qPCR offers the most sensitive profiling signal with a reasonable cost. With a considerate design, RT-qPCR can also achieve high throughput miRNA profiling. The recent progress of NGS has led to rapid cost reduction, which lowered the impact of NGS application in miRNA profiling. Nonetheless, microarrays with immobilized DNA probes on solid substrate are still the choice of many laboratories with a moderate budget [18]. Hence, microarrays are widely applied also in miRNA profiling [19]. One of the challenges of miRNA expression profiling is to distinguish between mature miRNAs and their precursors and to detect the miRNA expression signal with high specificity. This problem can be solved with a precise and genuine probe design on microarray platforms.

Much more attention has been dedicated to mature miRNA expression profiling, although pri-miRNA and pre-miRNA are important intermediates during miRNA biogenesis [20]. Pre-miRNAs have also been acknowledged as useful disease biomarkers [21,22]. RT-qPCR has been, so far, the gold standard for pre-miRNA expression evaluation [23]. Recently, a global pre-miRNA landscape has also been successfully provided by applying a deep sequencing technique [24]. Since pre-miRNAs and mature miRNAs can both be detected in cytoplasm, we demonstrated in this paper the possibility of exploring the expression of mature and pre-miRNAs on a single microarray platform, simultaneously. 

## 2. Experimental Section

**Figure 1:**
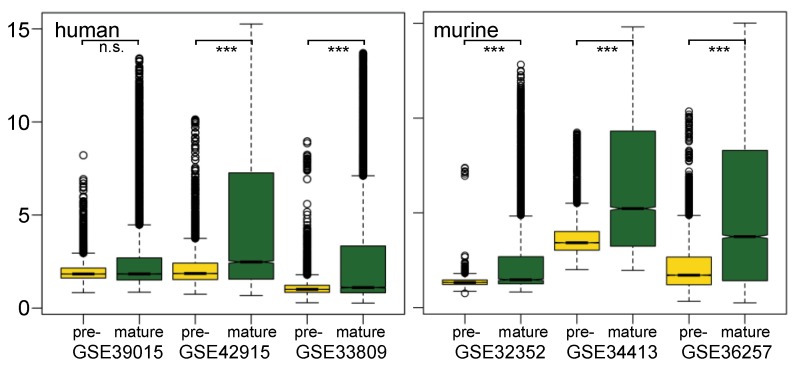
Distribution of normalized expression values of pre-miRNAs and mature miRNAs: shown are boxplots of miRNA expression values after normalization for all arrays in 3 human studies (left part) and 3 murine studies (right part). Yellow color represents pre-mature miRNA expression signals, and green color stands for mature miRNA expression signals. The ordinate shows transformed signal intensities in arbitrary units. Significance: n.s. = not significant; *** = significant, with a *p*-value < 0.001.

Affymetrix GeneChip^®^ miRNA 2.0 arrays (Affymetrix, Santa Clara, California, USA) contain probes that interrogate the mature miRNA and pre-miRNA of a wide spectrum of species. In this study, profiling data of human and mouse samples (GSE39015 [25], GSE42915 and GSE33809 [26] focused on human samples; GSE33413 [27], GSE32352 [28] and GSE36257 [29] profiled mouse samples) on Affymetrix GeneChip miRNA 2.0 arrays (see Table 1) were retrieved from the Gene Expression Omnibus (GEO) database [30]. Raw data were normalized with the robust multi-array average (RMA). Present calls were made by using the detection above background (DABG) algorithm with a *p*-value cutoff of 0.05. Pre-miRNA and mature miRNA probes were identified according to annotations provided by Affymetrix. A paired *t*-test was applied on the median expression of miRNAs and pre-miRNAs for significancy calculations (see Figure 1). 

**Table 1:** Information of data sets involved in this paper.

Study	Species	No. of samples	Short Description of the Study	RNA extraction method
GSE34413	*M. musculus*	18	whole brain tissue from day 70, fetal alcohol exposed males and matched controls	TRIzol (Invitrogen)
GSE32352	*M. musculus*	6	EC/NSPC co-cultures were incubated with FGF/VEGF receptor inhibitor and TGF-receptor inhibitor	mirVana miRNA (AB/Ambion)
GSE36257	*M. musculus*	24	heart, quadriceps femoris and diaphragm from 8-week-old male WT C57/B10 and male mdx C57/B10.	TRIzol (Invitrogen)
GSE39015	*H. sapiens*	18	2 cervix cell lines and 16 clinical tumor samples stored in FFPE	RecoverAll Total Nucleic Acid Isolation kit (Ambion)
GSE42915	*H. sapiens*	12	12 placentas (6 from first trimester and 6 from third trimester)	TRIzol (Invitrogen)
GSE33809	*H. sapiens*	24	SET2 cells were incubated with increasing concentrations of INC424/Ruxolitinib for 3-6 h	RNeasy Micro kit (Qiagen)

EC, endothelial cell; NSPC, neuro stem/progenitor cell; FGF, fibroblast growth factor; VEGF, vascular endothelial growth factor; TGF, transforming growth factor; FFPE, formalin-fixed, paraffin-embedded.

The correlation of pre- and mature miRNA expressions were studied under two different aspects: (i) for study, i, (i = a GEO study re-analyzed in this manuscript: GSE39015, GSE42915, GSE33809, GSE33413, GSE32352 and GSE36257), the expression values of pre-miRNAs can be described as *V_i_*_,pre_ = {*v*_1,pre; _*v*_2,pre;_ …; *v*_j,pre_|j = the array number in study i} and the expression values of mature miRNAs as *V_i_*_,mature_ = {*v*_1,mature;_* v*_2,mature;_ …; *v*_j,mature_|j = the array number in study i}. Pearson correlation coefficients were pairwise calculated between *V_i,_*_pre_ and *V_i,_*_mature_ for all arrays in studies, i, and then presented as a bar plot (see Figure 4). (ii) The expression matrix, M, of pre-miRNA and mature miRNA in study, i, was defined as *M*_i,pre_ = {*m*_1,pre; _*m*_2,pre;_ …; *m*_k,pre_|k = the number of pre-miRNAs on one array} and *M*_i,mature_ = {*m*_1,mature; _*m*_2,mature;_ …; *m*_k,mature_|k = the number of mature miRNAs on one array}. Correlation between *M*_i,pre_ and *M*_i,mature_ was also calculated by pairwise Pearson correlation coefficients. The distribution of pairwise correlation coefficients of all miRNAs in every study were shown as a histogram (Figure 5). Statistical analysis were completed with basic functions in R [31].

## 3. Results and Discussion

GeneChip^®^ miRNA 2.0 has 4,592 probe sets for human and 1,412 probe sets for mouse. Seven hundred sixty seven pre-miRNAs and 919 mature miRNAs are annotated for human samples. For the mouse samples, 510 pre-miRNAs and 601 mature miRNAs are available on the platform. Eight hundred thirty six pre-miRNA/mature-miRNA pairs can be mapped in human miRNA annotation, while there are 579 according to the mouse annotation. 

### 3.1. Mature miRNAs Are More Abundantly Detected

Pre-miRNAs showed lower expression in these data sets than their matured products. Boxplots of expression values of pre- and mature miRNAs in three human and three mouse studies, respectively, are shown (Figure 1). In all murine studies, median expressions of pre-miRNA are significantly lower than those of mature miRNA, wherein one of the studies exhibits a minor difference (GSE32352). For human samples, the differences are not so pronounced; however, they are also significant for two of the three studies analyzed (GSE42915, GSE33809). At the same time, the upper quartiles of mature miRNAs distribution are located more in a higher expression value region. It is possible that mature miRNA is more enriched by sample preparation. Besides, the high stability observed by mature miRNAs could also contribute to their higher expression signal [32]. Moreover, the rapid processing of the intermediate product pre-miRNA might have an impact on the fewer copy number of pre-miRNA compared to the stable accumulating signal of mature miRNA. The lower expression value of pre-miRNA compared to its corresponding mature miRNA was also reported in another study using the RT-qPCR method [33]. Independent to the variety of individual samples, three data sets that used the same RNA isolation kit (GSE34413, GSE36257 and GSE42915) showed a similar pattern in expression value distribution. This observation indicates that sample preparation, especially RNA isolation, is a crucial factor for successful miRNA profiling. 

### 3.2. Much More Mature miRNAs than Pre-miRNA Were Detected as Present

**Figure 2:**
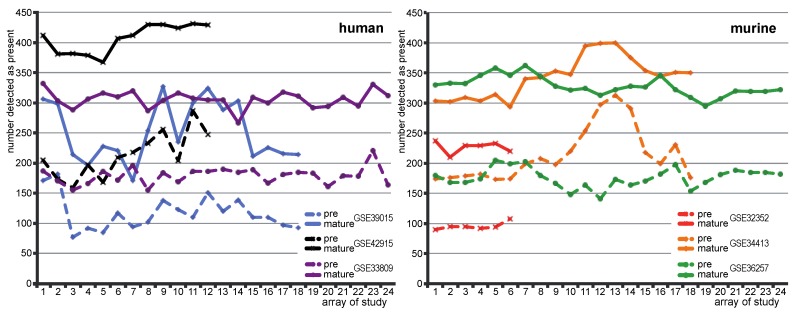
Number of pre-/mature miRNAs with present call in human (**left** panel) and murine studies (**right** panel): number of pre-miRNA and mature miRNA with present call on individual arrays in human and murine data sets. Dashed lines represent numbers of pre-miRNAs with a present call. Solid lines represent number of mature miRNAs with a present call. Colors of the lines distinguish different studies. Numbers on the abscissa indicate the numbers of arrays involved in corresponding data set.

The general higher expression level of mature miRNA was also reflected in the detection call of present miRNAs. Present calls were made for pre-miRNA and mature miRNAs compared to background signals. Mature miRNAs have almost doubled present calls with respect to pre-miRNAs in all data sets (Figure 2). 

**Figure 3:**
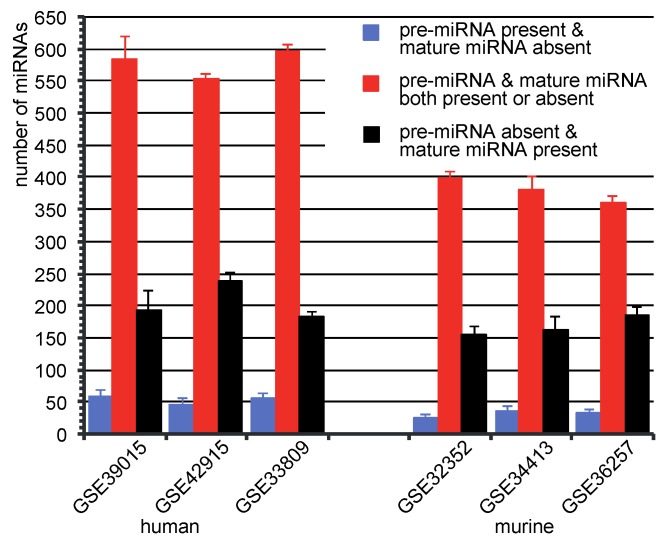
Number of miRNAs in three categories: average number of miRNAs with the standard deviation in the category, (i) pre-miRNA is called as present, while corresponded mature miRNA is called absent (blue bars), (ii) pre-miRNA and mature miRNA are called both present or absent (red bars) and (iii) pre-miRNA is called as absent, but mature miRNA is called present (black bars).

Most of the pre-miRNAs and mature miRNAs share the same present/absent calls. That means, present pre-miRNAs have present mature counterparts, and absent pre-miRNAs have also absent mature corresponding miRNAs (Figure 3, red bars). However, there are also present pre-miRNAs, which have absent mature miRNA, and *vice versa* (Figure 3, blue and black bars). miRNAs that were detected as present in mature form, but as absent in pre-miRNA form, could support the hypothesis that mature miRNAs are more stable than their precursor counterparts. As demonstrated, there was a few number of miRNAs that were present in their premature form, but absent in their mature form. This phenomenon indicates a possible miRNA transcription regulation by degrading mature miRNA [34]. The reduction of specific mature miRNAs compared to their pre-miRNAs was also reported in human colorectal neoplasia by Northern analysis [35]. 

### 3.3. Correlation between Pre-miRNA Expression and Mature miRNA Expression

**Figure 4:**
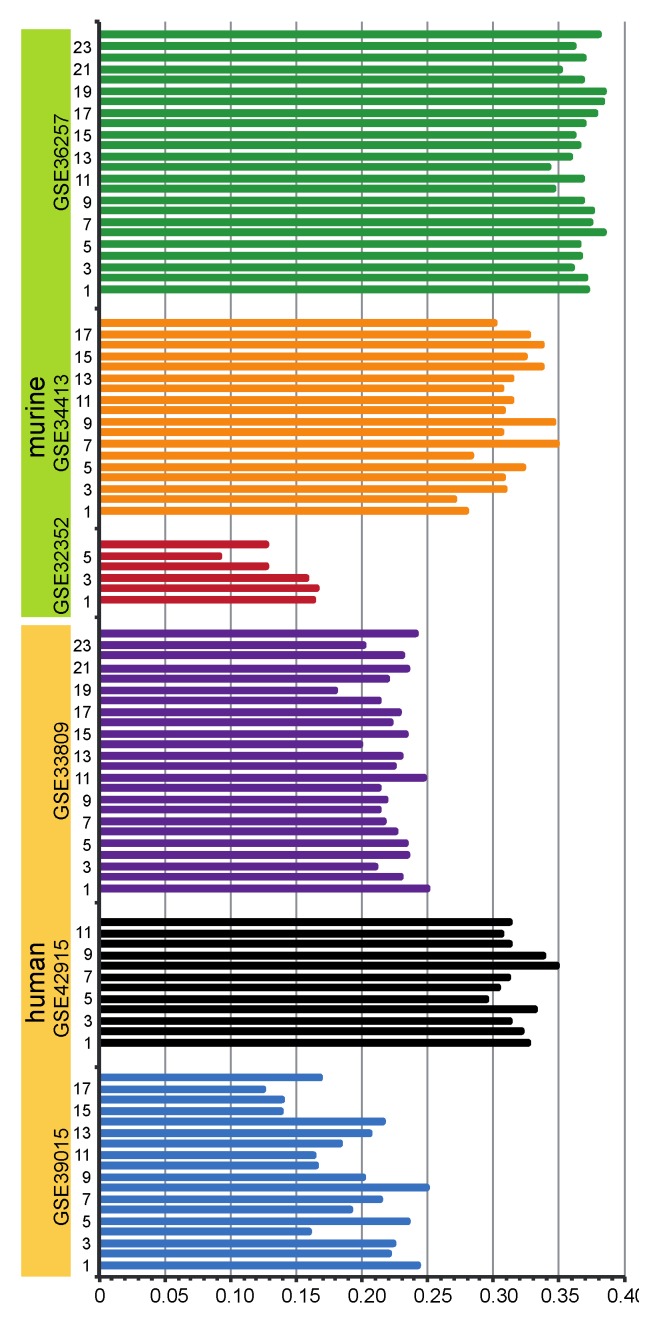
Correlation of pre-/mature miRNA expression: shown are the correlations of pre-miRNA and mature miRNA expression on the same arrays of murine (upper part) and human **(**lower part) data sets. The abscissa indicates correlation coefficients. In the ordinate axis, the number of arrays involved in the corresponding study are shown.

The expression of mature miRNA and pre-miRNA measured on the same arrays showed a positive interdependency (Figure 4). Notwithstanding different expression levels, a positive correlation did exist between expression of pre- and mature miRNAs on the same array. This is consistent with the results reported previously [36]. Correlation coefficients of pre- and mature miRNA on the same array in one data set were consistent, while the values clearly varied between different data sets. For example, GSE32352 demonstrated a much lower correlation coefficient between pre- and mature miRNA expression. GSE34413, GSE36257 and GSE42915 showed similar correlation coefficients. While in the studies, GSE34413, GSE36257 and GSE42915, the same RNA isolation kit (TRIzol from Invitrogen) was used; in the GSE32352 study, the mirVana RNA isolation kit (Ambion) was used. For this reason, we believe that the observed correlation coefficients were strongly affected by the choice of sample preparation. The influence of the kind of probe labeling can be excluded, because all probes were labeled with the same kit (FlashTag biotin HSR kit from Genisphere).

**Figure 5:**
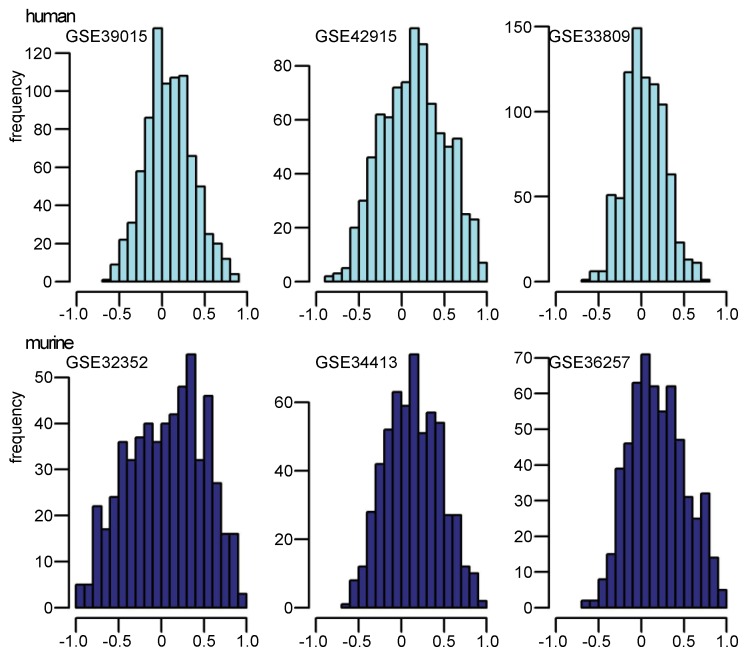
The correlation of pre-/mature miRNA expression according to expression pattern: histograms of correlation coefficients of all pre-miRNA and its corresponding mature miRNA expression patterns in three human (upper part with light blue bars) and three murine (lower part with blue bars) studies. Abscissae indicate correlation coefficients.

Correlation coefficients were also calculated for the expression profile of every mature miRNA to its corresponding pre-miRNA across the arrays in the data sets. These coefficients support the evidence of the dependency between pre-miRNA and mature miRNA expression regulation, which differs according to different tissue sources and/or treatments. Correlation coefficients for all miRNAs in each study have been calculated between the pre-miRNA expression pattern and the mature miRNA expression pattern (Figure 5). In all data sets analyzed in this paper, correlation coefficients were widely distributed between −1 and 1. In particular, not only positive coefficients were observed, but also extreme negative ones. That means that upregulation of mature miRNA can be observed in a study even when the corresponding pre-miRNA is downregulated. This result indicates that during maturation of pre-miRNA to mature miRNA, complex factors are involved in regulation, depending on the kind of treatment, tissue-specific development or different biological/pathological contexts.

## 4. Conclusions

Six publicly available miRNA profiling raw data sets generated from human and mouse samples on Affymetrix GeneChip^®^ miRNA 2.0 array platforms were re-analyzed in this study. Pre-miRNA and mature miRNA expression signals were retrieved and normalized to gain comparable signals. miRNA precursors exhibited a lower expression level in most of the analyzed data sets. Present calls of individual mature miRNAs and pre-miRNAs showed a great part consistency, with minor discrepancies. On the same array, mature miRNA expressions are positively correlated to pre-miRNA expressions. The distribution of expression values of pre-miRNA and mature miRNA, as well as the correlation coefficients between them, seem to be influenced by the RNA isolation methods applied in the studies. Because of the sample size limitation and the lack of other experimental verifications, we are not able to judge the pros and cons of RNA isolation methods for miRNA profiling at this point. 

The expression regulation patterns of mature miRNAs showed no clear positive correlation to the expression regulation patterns of the corresponding pre-miRNAs. Therefore, we believe in the existence of regulation factors of miRNA maturation according to treatment, tissue type and biological/pathological contexts involved in individual studies. The results presented in this paper require definitely further research and verification with more data sets and with other techniques, like RT-qPCR. Nevertheless, our study demonstrated the possibility of profiling pre-miRNA and mature miRNA simultaneously without separate sample preparation. Compared to methods like RT-qPCR, the microarray platform applied in this paper has the advantage of data normalization without the bias caused by specific reference RNA(s). At the same time, we can obtain a genome-wide overview for pre-miRNA and miRNA expression. An additional advantage of this platform is that only one simple labeling procedure is sufficient for both pre-miRNA and mature miRNA profiling. 
