# *Correction*: Gan, L.; Denecke, B. Profiling Pre-MicroRNA and Mature MicroRNA Expressions Using a Single Microarray and Avoiding Separate Sample Preparation. *Microarrays* 2013, *2*, 24-33

**DOI:** 10.3390/microarrays2030170

**Published:** 2013-06-24

**Authors:** Lin Gan, Bernd Denecke

**Affiliations:** Interdisciplinary Centre for Clinical Research Aachen, RWTH Aachen University, Pauwelstr. 30, 52074 Aachen, Germany; E-Mail: lgan@ukaachen.de

It came to our attention that a paper has recently been published concerning one of the GEO datasets (GSE34413) we cited in our published paper [1]. The original reference (reference 27) cited for this dataset leads to a paper about a similar study from the same research group [2]. In order to provide readers with exact citation information, we would like to update reference 27 in our previous paper to the new published paper concerning GSE34413 [3]. The authors apologize for this inconvenience.
